# Review of heterogeneous material objects modeling in additive manufacturing

**DOI:** 10.1186/s42492-020-0041-6

**Published:** 2020-03-05

**Authors:** Bin Li, Jianzhong Fu, Jiawei Feng, Ce Shang, Zhiwei Lin

**Affiliations:** 1 State Key Laboratory of Fluid Power and Mechatronic Systems, College of Mechanical Engineering, Zhejiang University, Hangzhou, 310027 China; 2 Key Laboratory of 3D Printing Process and Equipment of Zhejiang Province, College of Mechanical Engineering, Zhejiang University, Hangzhou, 310027 China

**Keywords:** Review, Heterogeneous objects modeling, Heterogeneous materials, Additive manufacturing

## Abstract

This review investigates the recent developments of heterogeneous objects modeling in additive manufacturing (AM), as well as general problems and widespread solutions to the modeling methods of heterogeneous objects. Prevalent heterogeneous object representations are generally categorized based on the different expression or data structure employed therein, and the state-of-the-art of process planning procedures for AM is reviewed via different vigorous solutions for part orientation, slicing methods, and path planning strategies. Finally, some evident problems and possible future directions of investigation are discussed.

## Background

In contrast to traditional engineering objects with uniform materials, heterogeneous material objects, which refer to objects that comprise spatially different material compositions or structures, have gained considerable research interest. Multi-material objects, objects with functionally graded material (FGM) distributions, micro-electro-mechanical systems devices, porous structures, and assembly composites are typical examples of heterogeneous material objects [1]. In this study, we focus on multi-material objects and objects with FGM distributions for convenience. Multi-material objects are associated with discrete material distributions, where finite integer indexes are typically used to represent different homogeneous materials; whereas FGM-based objects are characterized by gradually varying material properties.

In heterogeneous material objects, which are believed to possess superior properties, several contrasting materials are incorporated into a single object to render it suitable for use in special applications, such as mechanical [2, 3], biomedical [4, 5], energy [6, 7], optical [8, 9] and other fields. By designing anisotropic properties incorporates with material heterogeneities, the advantageous properties of various materials can be fully exploited; further, traditional limitations due to material incompatibilities can be naturally overcome with gradually varying material properties.

The current developments in additive manufacturing (AM) techniques enable the formation of complex geometric structure with customized material properties. Intuitively, if the composition of a material changing in different locations within a product, this technique would exhibit significant potential for fabricating heterogeneous material objects.

There are varieties of AM techniques for fabricating heterogeneous material objects [10]. These techniques differ from each other in terms of the compatibility of the type of materials used and manufacturability restrictions imposed by the machine. Stereo-lithography (SLA) [11, 12] produces parts by solidifying each layer of photopolymer liquid resin via an ultraviolet laser. The material distribution is homogeneous in each layer, but could be changed along the build direction. The fabricating principle of fused deposition modeling (FDM) technique [13, 14] is to extrude filaments of molten thermoplastics material through heated nozzles. When heated nozzles allow for an arbitrary mixture of different filament materials, FDM devices with multiple nozzles can additively fabricate FGM-based objects. Laser-based AM techniques [15, 16] could fabricate heterogeneous metal parts with remarkable structural strength, surface roughness, and manufacturing precision depending upon the type of machine and materials used. By jetting layers of curable liquid photopolymer, Polyjet 3 dimensional (3D) printing [17] obtains the continuous gradient profile, which offers exceptional detail, surface smoothness, and precision. In contrast to traditional manufacturing techniques, these heterogeneous material printing techniques offer the capabilities of cost-effective automation of fabrication process and provide greater flexibility to design the complex distribution of heterogeneous materials locally.

Moreover, these heterogeneous material AM techniques can fabricate objects that were previously impossible or exceedingly difficult to manufacture; further, these methods also allow the mass customization of personalized products. A multi-material 3D printing platform was developed in ref. [18], which integrates machine vision with 3D printing, simplifies the overall platform design, and enables extensible applications over auxiliary parts with a high resolution and a low cost. To fabricate a roly-poly toy, Zhao et al. [19] provided a novel and straightforward method that designs the toy using a computer-aided method and fabricates it through an ordinary 3D printing machine. Inspired by traditional Chinese sugar painting art, Zhao et al. [20] utilized a tailor-made 3D food printer to present a novel personalized food-printing framework driven by portrait images. The AM techniques have also motivated significant research effort in the field of robotics; the first multi-axis 3D printing approach was presented in ref. [21], which enabled fabrication of general volume models along the variable directions with minimal supporting structures. The realization of increasingly personalized applications will not only contribute toward significant future research in AM, but also inspire various, as yet unexplored, applications in industrial markets.

Based on different aspects of heterogeneous material objects, numerous review papers have been published, such as heterogeneous objects modeling [1], the design methods to improve functional performance [10], and the fabrication of FGM objects through AM techniques [22, 23]. In particular, the application of heterogeneous material objects via AM technologies should be systematically addressed in term of both the modeling techniques and the corresponding fabrication process. Therefore, the key contribution in this review is to provide an overview of the state-of-the-art heterogeneous material objects in AM.

The rest of this study is organized as follows. Section 2 summarizes the representation and modeling techniques for heterogeneous material objects, and section 3 discusses the main process planning for the fabrication of heterogeneous material objects via AM techniques. In the last section, the conclusion and some evident problems have been discussed.

## Representation approaches of heterogeneous objects

Conventional computer aided design (CAD) modeling methods focus on representing the geometry and topology of an object, but material information is not easily available within these representations. This poses a great limitation for the downstream applications of heterogeneous material objects. A generic and systematic modeling scheme for the design, analysis, and fabrication of heterogeneous material objects is crucial to leverage the full capabilities of heterogeneous material objects. Important attributes for representing heterogeneous objects include geometrical structure, material distribution, microstructure, tolerances, and operating condition. In this study, we focus on the fundamental attributes, geometrical structure and material distribution, for representing heterogeneous objects. A large and growing body of literature has investigated the extension of conventional geometric representation schemes, each with its pros and cons, to heterogeneous material objects.

### Voxel based model

Just as a pixel represents a 2 dimensional (2D) element in raster graphics, each voxel in volume graphics denotes a 3D element with a corresponding material distribution, and a collection of voxels could represent a heterogeneous material object as a voxel-base model. By acquiring medical data from computerized tomography (CT) and magnetic resonance imaging devices, medical images are such typical voxel-base examples. For 2D images, voxels are discrete blocks analogous to the rectangular pixels and represent such voxel-based model as a stepped bitmap. For 3D objects, a voxel-based model is represented as a 3D matrix, and each element of the matrix corresponds to a voxel. The simplest form occurs when the voxels assume values as one or zero, with one donating a solid element and zero donating a void element in space.

**Fig. 1:**
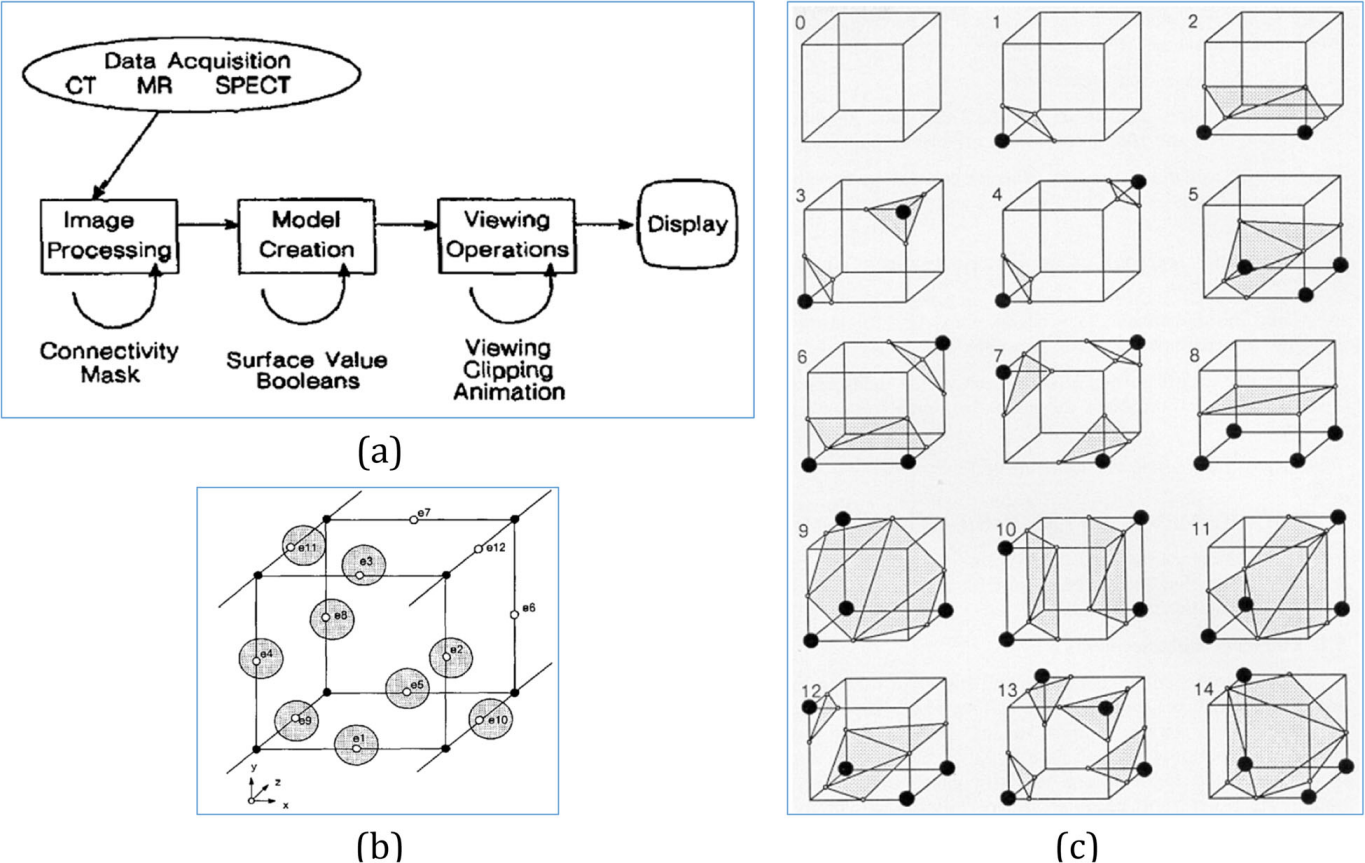
The 3D medical information flow (**a**), interpolation process (**b**) and defined templates (**c**) in Marching Cubes techniques [25]

In volume graphics, the representation of voxel-based objects is an indispensable stage, called voxelization [24], that concerns with best approximates from general continuous geometric representation into a set of discrete voxels. Just as the scan-conversion process that pixelizes 2D objects, there also refers to a 3D scan-conversion process. As shown in Fig. 1, the Marching Cubes technique [25] is a 3D surface construction algorithm with high-resolution that creates a polygonal representation of constant-density surfaces from medical images. To scan-convert 3D planar polygons into their discrete voxel-map representation, Kaufman [26] presented a 3D scan-conversion algorithm within a cubic frame buffer. Contrast to constructive solid geometry (CSG), which cannot express the interior description of an object, an algebraic scheme called constructive volume geometry [27] represented complex volume objects as voxel-based models. By using combinational operations in this scheme, each voxel is associated with a uniquely determined scalar value via an interpolation function.

Based on volumetric rendering schemes by texturing in computer graphics, FGM distributions were designed for the creation of a material cloud in a confined geometric space and in a structured and controlled manner [28]. Under the given thermal loading and boundary conditions, the thermoplastic behavior of heat-resisting FGMs was characterized by the spatial distribution of volume fractions of constituent particles [29]. Following this, a 2D volume-fraction optimization procedure was addressed for relaxing the effective thermal stress distribution, where the material properties were input through Gauss integration points. A detailed approach [30] was developed for determining the optimal material properties required in different locations of a component made of multi-heterogeneous materials. This design process was reversed, i.e., from its functional requirements, to material properties, and to constituent compositions.

**Fig. 2:**
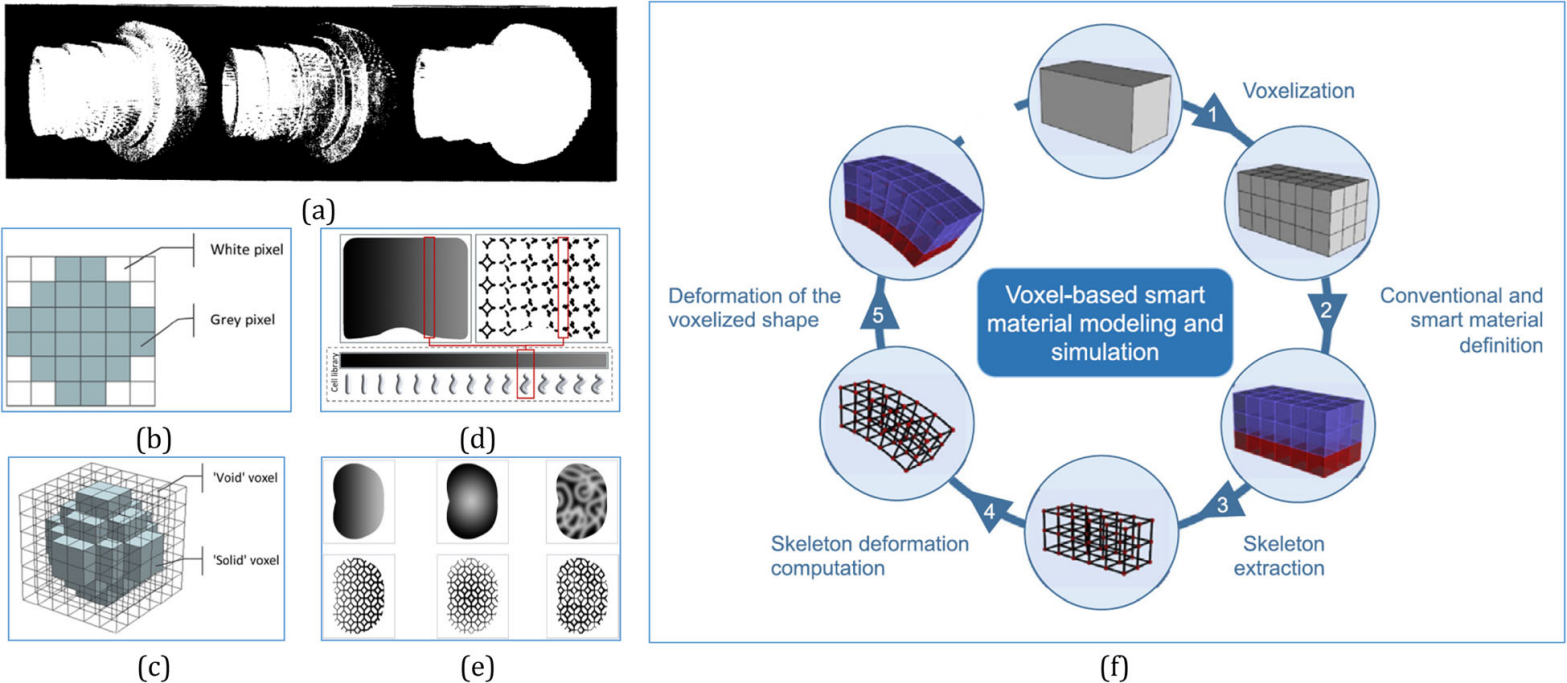
**a** Object voxelized along the slicing direction based on three different resolutions [31]; A voxel-based method of constructing and skinning functionally graded lattice structures suitable for AM [32]: pixel image of a circle with a low resolution (**b**); voxel model of a sphere with a low resolution (**c**); a domain with functionally grading gradients (**d**); functionally graded structures generated from input gradients (**e**). **f** The conceptual design framework for 4D printing [33]

Among the current AM techniques, the voxel-based approach is well suited to parallel systems. In ref. [31], masks are used to create successive layers of the component, and then a light source is utilized to solidify materials on surfaces. As shown in Fig. 2a, during manufacturing along the slicing direction, the resolutions of the voxelization could correspond to the layer thickness and manufacturing accuracy. During fabrication, as each voxel could be precisely translated as a quantifiable unit with deposited material information, this measure reasonably estimates the accurate actual volume of the object, and is somehow independent of the topological structure.

To address lattice structures combined or embedded within a complex geometrical structure, a voxel-based method [32] was proposed for the generation of trimmed lattice structures. As shown in Fig. 2b-e, these trimmed lattice structures are highly flexible in terms of both lattice cell type and external geometry, and more suitable to fabricate highly complex lattices via AM techniques. As a new breakthrough in AM, 4 dimensional (4D) printing becomes another rapidly growing manufacturing technique, which is an AM technique with smart materials. The modeling framework in Fig. 2f was proposed for simulating smart materials and conventional materials behaviors on a voxel basis [33], which allows for arrangement of material distributions and rapid evaluation of the corresponding behaviors. With this modeling framework, designers are provided with the possibility of testing a given distribution of smart materials rapidly and checking how its behaviors expose to stimulus before proceeding into detail design.

### Volume mesh based model

In a manner similar to the voxel-based modeling techniques, mesh-based modeling could assign local material component information to each element existing in the finite-element-based representation. In term of volume mesh based model, a heterogeneous object is represented as a collection of polyhedrons; each polyhedron is represented as a list of vertices, where geometric position as well as the material compositions information can be stored.

**Fig. 3:**
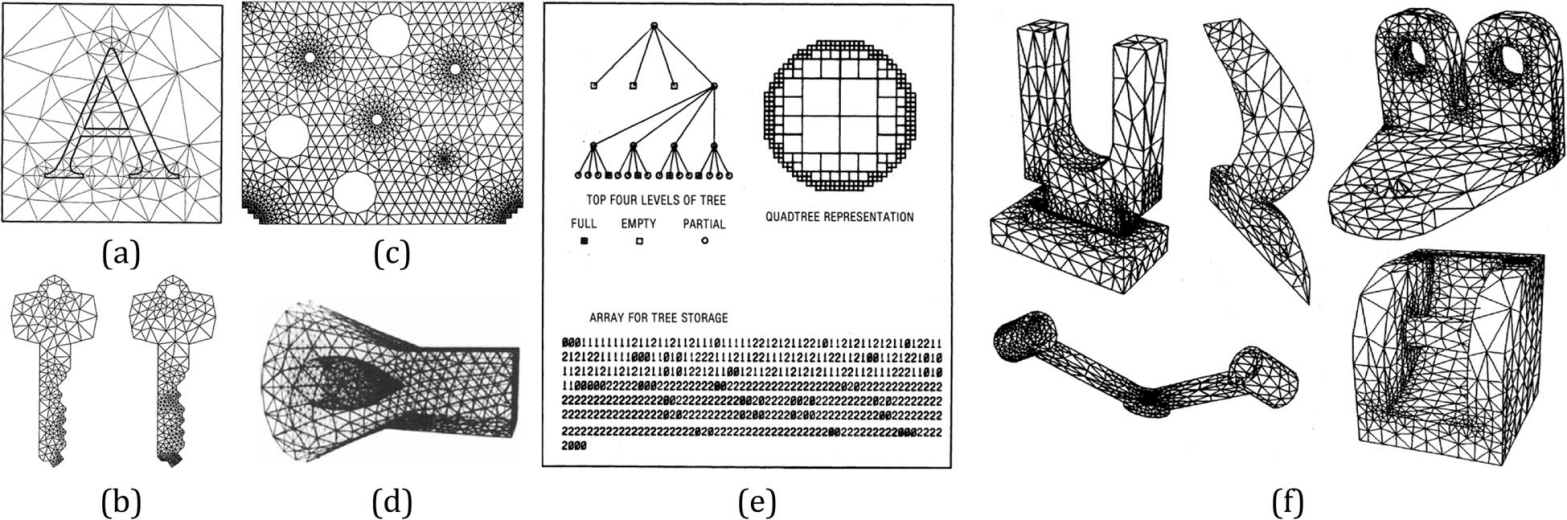
**a** Triangulation produced by the Delaunay refinement algorithm with minimum angle ≈20^°^ [35]. **b** Based on a minimum angle ≈ 33^°^, the left mesh is formed by always splitting the worst existing triangle, and the right mesh is formed by using a first-come first-split queue of bad triangles [36]. **c** Based on a domain with many holes, refined meshes can be progressively generated around points of interest [37]. **d** 3D unstructured grids generated by the advancing-front method [38]. **e** The finite element mesh of a circle generated via quadtree representation, [39]. **f** Automatic 3D mesh generation by the modified-octree technique [40]

**Fig. 4:**
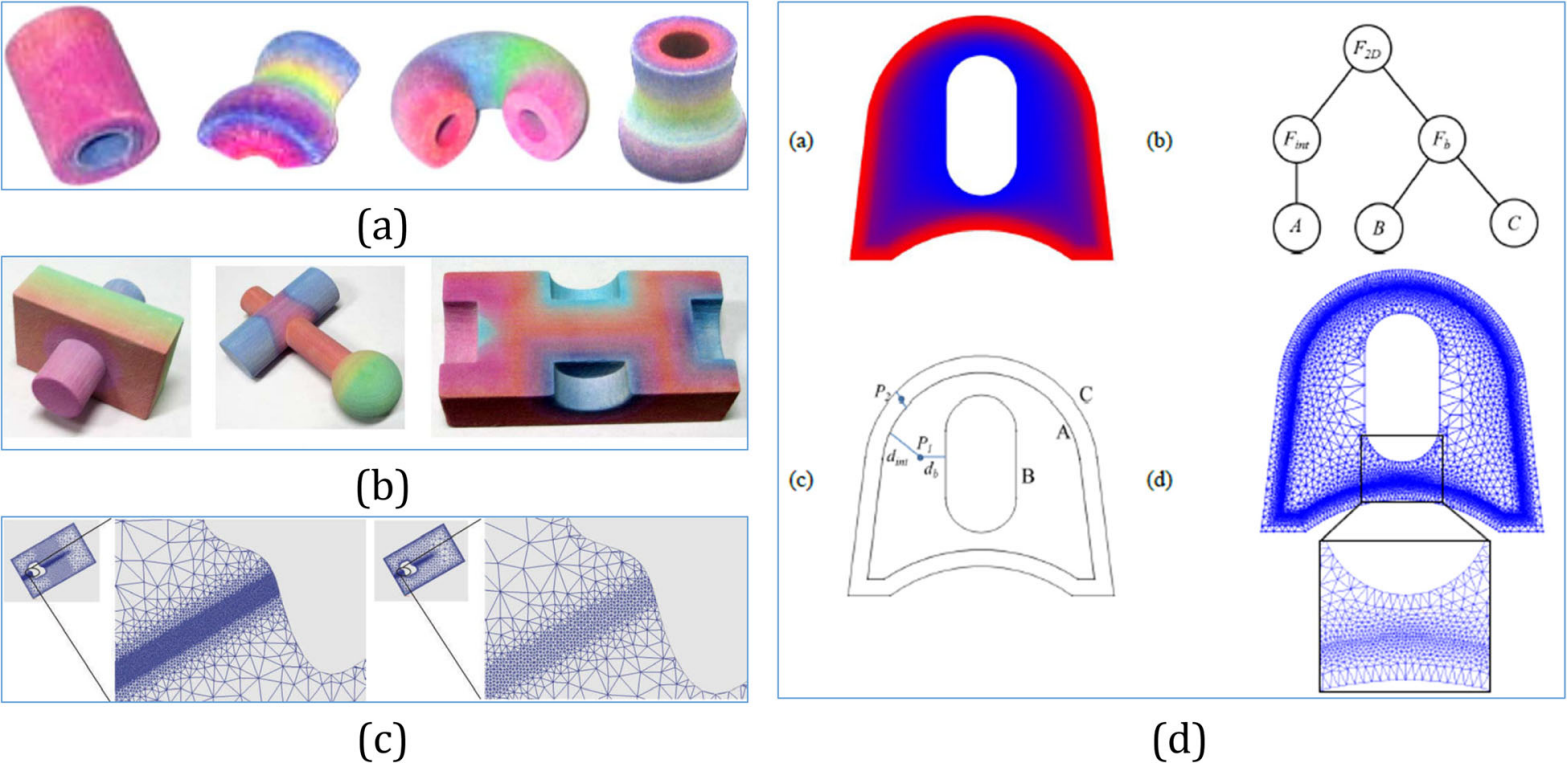
**a** Prototypes fabricated with a hierarchical representation for unidirectional material gradient [45]. **b** Prototypes fabricated with non-manifold heterogeneous cells [46]. **c** Adaptive meshing results of 2D heterogeneous objects using material quadtree based on different thresholds [47]. **d** Heterogeneous feature tree structure processes objects with complex material gradient functions [48]

Based on the finite element analysis (FEA), automatic mesh generation schemes for objects with homogeneous materials have been extensively studied. The basic principle of these schemes is to construct adaptive meshes with regard to a node spacing function [34], as shown in Fig. 3. The Delaunay triangulation methods [35, 36], advancing front methods [37, 38], Quadtree and Octree methods [39, 40] are the most commonly used methods. These methods mainly take the compatibility of the geometric and topological structure into consideration [41]. Therefore, adaptive meshes with heterogeneous materials have gained widely investigation. However, most of them did not consider the local material heterogeneities, as the meshes are typically generated as the extension of homogeneous solid modeling approaches [42–44]. A computationally efficient work done in ref. [45] was the first to study the adaptive meshing purpose for FGM objects. In this study, continuous material gradation was converted into step-wise variation, and a triangular mesh was generated inside each isomaterial region. However, as shown in Fig. 4a, only unidirectional material gradient is considered. As shown in Fig. 4b, a more generic method was proposed to generate adaptive mesh with bidirectional or even more complex material distributions [46].

An adaptive mesh generation method was proposed based on the quadtree technique for modeling objects with complex heterogeneous materials [47]. As shown in Fig. 4c, the subdivision of the domain was recursively processed until all the elements satisfied the material threshold requirement. The heterogeneous feature tree structure [48] shown in Fig. 4d also could process objects with complex material gradient functions and generate robust adaptive meshes. In ref. [49], a simple analytic function-based data representation was implemented to provide a generic solution suited for the mesh generation of heterogeneous materials. It generates adaptive meshes for general heterogeneous models efficiently without introducing unnecessarily dense meshes.

**Fig. 5:**
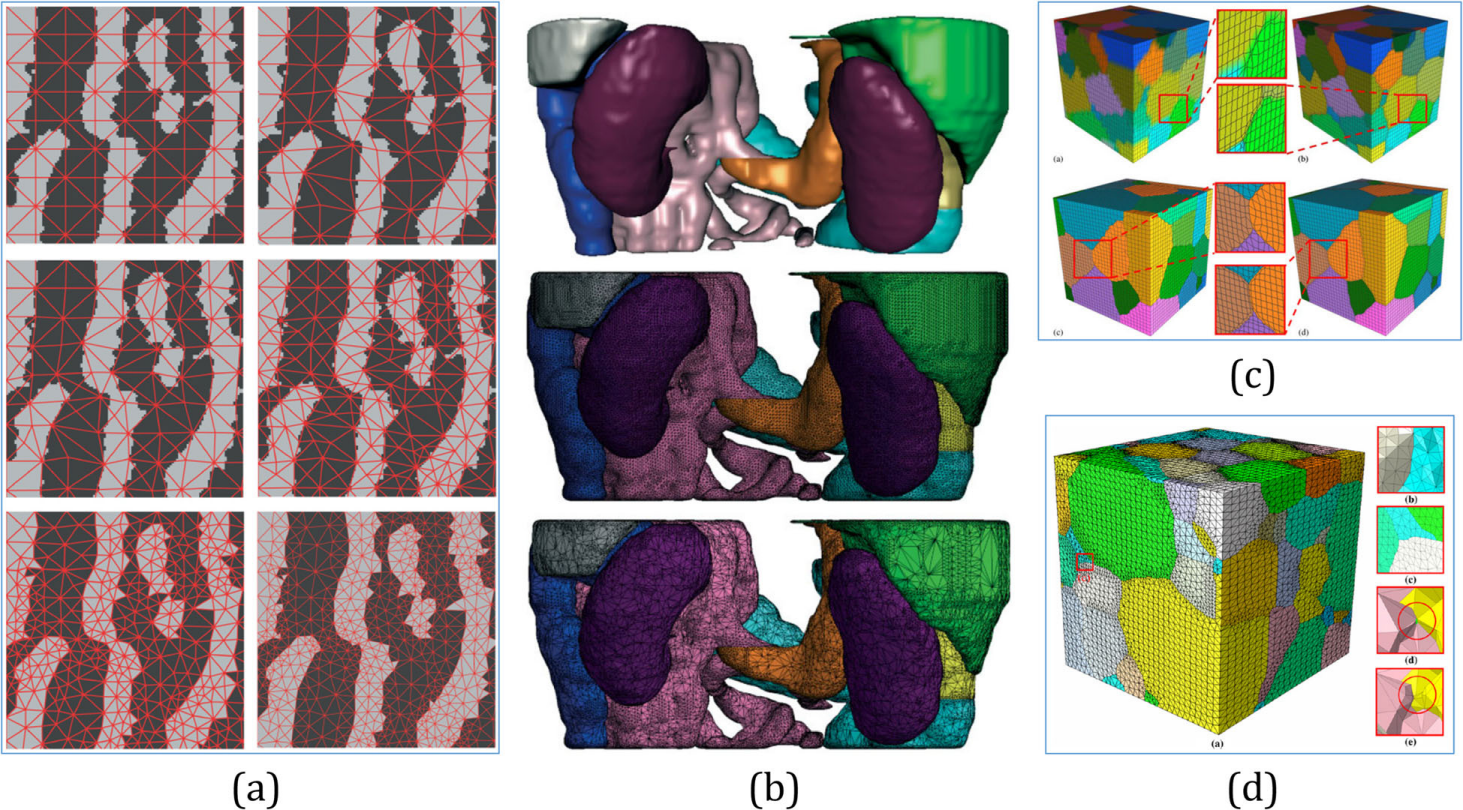
**a** Steps in the meshing process of material microstructure employing an image-based FEA [50]. **b** Surface mesh results before and after decimation using the multiple material marching cubes algorithm [51]. **c** Non-manifold hexahedral meshes before and after quality improvement for the critical feature determination of microstructure materials [52]. **d** Adaptive triangular and tetrahedral meshes of a 308-grain polycrystalline material upon resolving topology ambiguity [53]

For objects with complex microstructure, determining a material’s macroscopic properties given its microscopic structure, is of fundamental importance to materials science. As shown in Fig. 5a, two public-domain programs were described in ref. [50], which could jointly predict macroscopic behavior. These programs start from an image of the microstructure and end with results from finite-element calculations. As shown in Fig. 5b, for objects with multiple materials in computational biomedical engineering, an innovative system was proposed in ref. [51], which efficiently reconstructs multiple-material 3D surface meshes from 2D medical images. The enhanced multi-material marching cubes algorithm could extract boundary surfaces between different material domains within one sweep of the image stack in an integrated manner, and ensure the continuity and integrity of the surfaces. To deal with composite domain made up of heterogeneous materials, an automatic and efficient approach was proposed in ref. [54] to construct unstructured tetrahedral and hexahedral meshes, where the boundaries of these material regions form non-manifold surfaces. As shown in Fig. 5c, a novel approach was proposed in ref. [52] as the extension in ref. [54] to improve the quality of non-manifold hexahedral meshes further with feature preservation for microstructure materials. When categorizing all the vertices into seven groups, a comprehensive method processes with pillowing, geometric flow and optimization techniques to improve mesh quality. To generate quality triangular and tetrahedral meshes further, a novel approach was developed in ref. [53], which is an automatic and efficient approach to resolve topology ambiguity for multiple-material domains. As shown in Fig. 5d, this method could resolve topology ambiguity by analyzing a hybrid octree with both cubic and tetrahedral leaf cells, and be suitable for both homogeneous and multiple-material domains.

### Control point based model

Traditional modeling methods, such as boundary representation (B-rep) and CSG, are not the most suitable methods to use for heterogeneous objects. This is because they do not allow representation of the inside of the object precisely. With additional material information assigned to each of the control points, the control point based model can be regarded as a direct extension of parametric curves, surfaces and volumes.

During the 1980s, a tool for modeling heterogeneous objects was developed by using hyperpatches [55]. Owing to its high degree of freedom, this mathematical tool was useful to represent interior object by assigning the desired material information to every point. Then, a new Bézier hyperpatches based framework for modeling heterogeneous objects was proposed in ref. [56]. This framework defines an adequate mathematical model that characterizes heterogeneous objects precisely and represents the modeled objects as a computational representation.

**Fig. 6:**
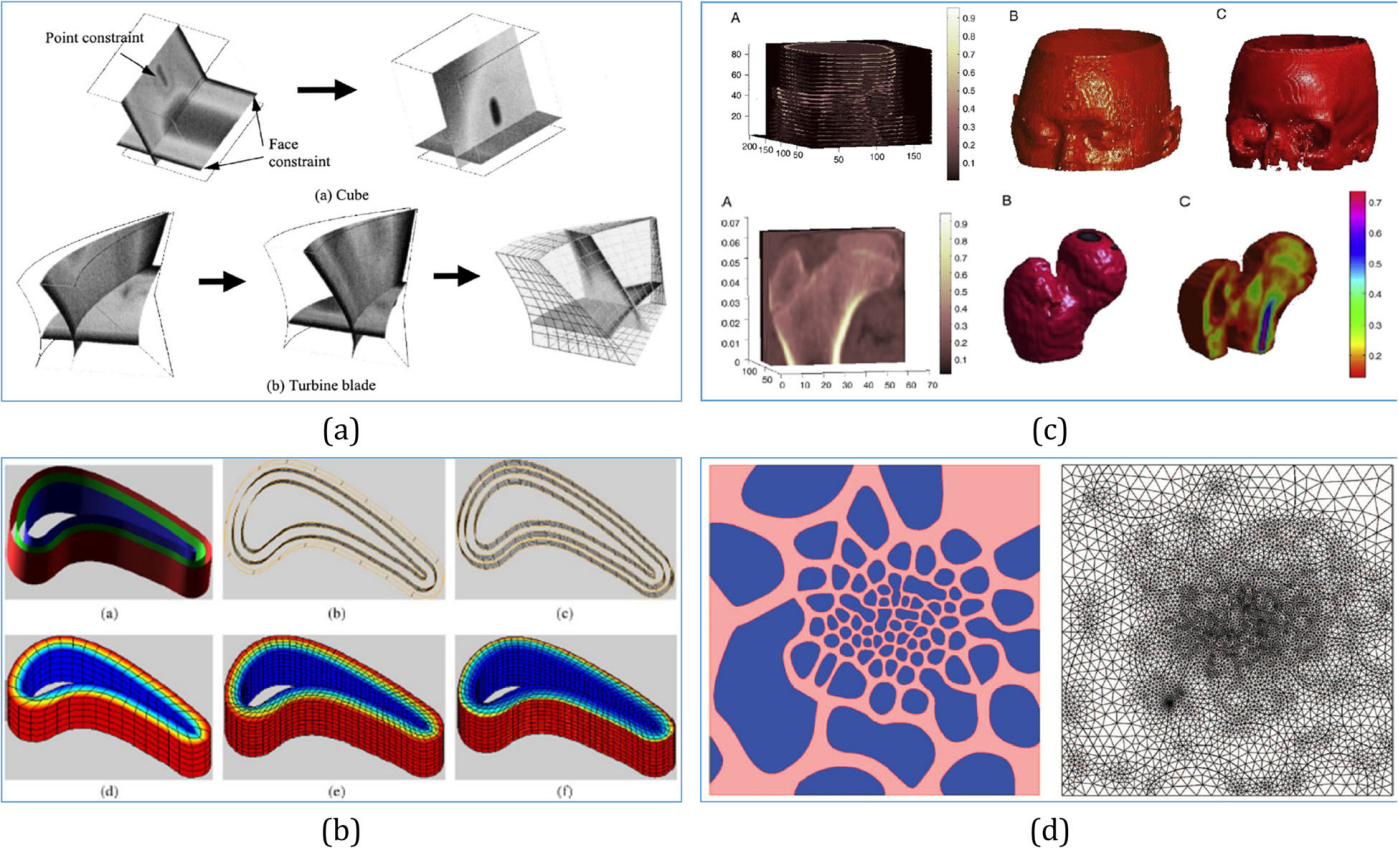
**a** A B-spline-hyperpatches-based heterogeneous object modeling with diffusion equations [57]. **b** A B-Spline-based approach to 3D conduction analysis for heterogeneous turbine blade via heterogeneous lofting [58]. **c** Using regular B-spline hyperpatches to accommodate the femur data and adjust the distribution values in the control mesh [59]. **d** Microstructural model of an FGM with graded volume fractions based on the stochastic Voronoi diagram and B-spline representation [60, 61]

Combining with the B-spline, a B-Spline-hyperpatches-based method was proposed to represent heterogeneous objects [57], which defined a diffusion process to assign the desired materials to each point of the object. By using B-spline hyperpatches, a lofting process from curves was proposed in ref. [58]. As shown in Fig. 6b, this method could be a good approach for modeling heterogeneous objects under certain problems. To represent the human body from CT scanned data in Fig. 6c, regular B-Spline hyperpatches were used to accommodate the scanned slices and adjust the distribution values in the vertices of the B-spline control mesh [59].

For heterogeneous objects with microstructures, it is necessary to retain the irregular topology or complex network in nature, and design a generic method for downstream applications, such as model validations, simulations, and microstructural optimizations. Based on the stochastic Voronoi diagram and B-spline representation, Kou et al. [60] proposed a novel digital model to design generic porous structures with graded porosities and irregular pore distributions. As shown in Fig 6d, Kou et al. [61] proposed a new method to model the microstructures with FGM distributions. As the extension of ref. [60], this approach does not require expensive imaging equipment during the design process, and digital models can be constructed at interactive or quasi-interactive rate.

**Fig. 7:**
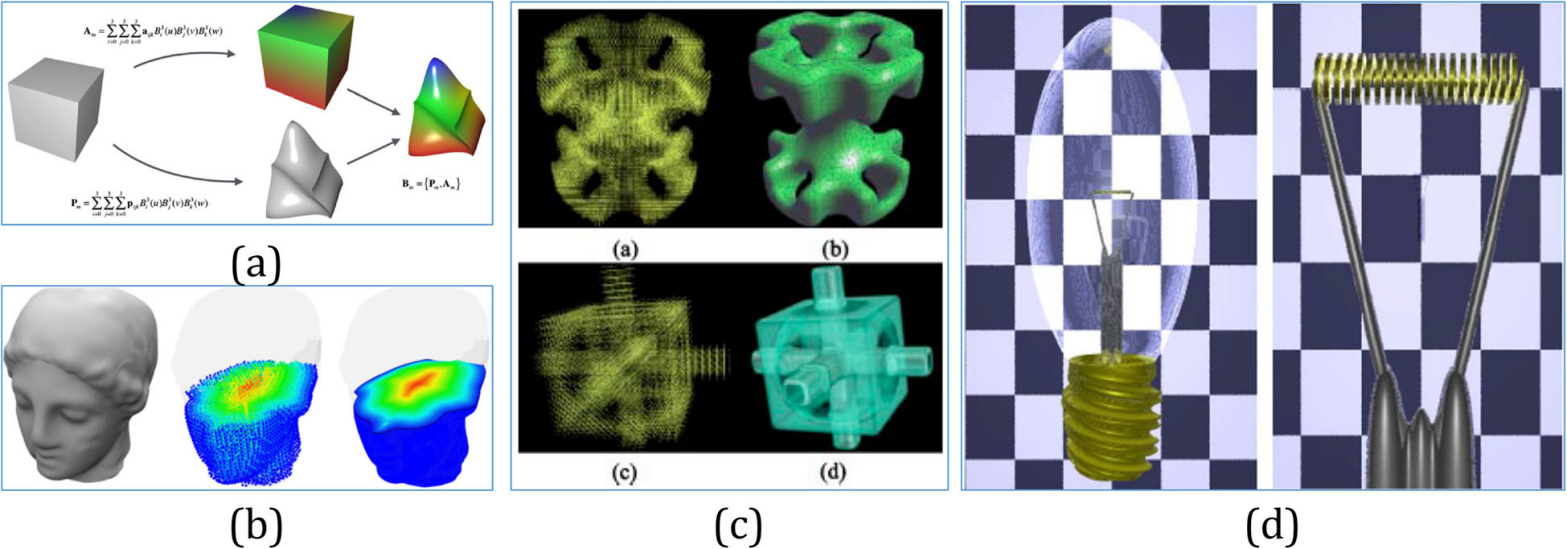
**a** A comprehensive framework for modeling heterogeneous objects [56]. **b** Volumetric attribute data represented by trivariate B-spline functions [64]. **c** Trivariate Simplex Splines for inhomogeneous solid modeling [65, 66]. **d** Complex heterogeneous object modeled using trivariate B-spline primitives [67]

With the advent of isogeometric analysis (IGA), the modeling of spline solids became an important topic. In ref. [62], a volumetric representation was proposed for geometric modeling based on trimmed B-spline. With these capabilities, the proposed framework can support volumetric IGA needs and can represent and manage heterogeneous materials for AM. Based on subdividing the solid model into sub-regions and associating analytic composition blending functions with each region in ref. [63], the blending functions defined the composition throughout the model as mixtures of the primary materials available to the solid freeform fabrication machine. Furthermore, as shown in Fig. 7c, a model based on trivariate simplex splines was presented in refs. [65, 66]. As the starting point, this representation is constructed using a triangle mesh, which allows for obtaining very accurate tetrahedral spline decompositions. In this way, the generated simplex B-spline mesh was huge, even for objects with relatively simple geometries. As shown in Fig. 7d, when modeling heterogeneous objects combining with trivariate B-splines primitives throughout CSG operations, a useful approach for rapid prototyping was proposed in ref. [67].

A mathematical framework was introduced using different trivariate volumes to represent the geometries and attributes independently [68]. In this framework, the control mesh of the attribute model can be different from the geometry model, while they share the same parameter domain. In this way, a simple geometry with complicated attributes, and vice versa, can be represented with the desired accuracy. By using the framework in Fig. 7a, a scheme for the modeling of heterogeneous objects via trivariate B-spline functions was proposed in ref. [64]. Then, the B-spline volume and the associated attribute data are directly sliced without converting them to SLA format, resulting in a tool path with fewer errors. In ref. [69], Catmull–Clark solids were utilized to model heterogeneous objects and were then sliced as printable bitmaps for multi-material AM techniques.

### Implicit function based model

Implicit function based models use the functional representation (F-Rep) [70] as the basic model for both the point set geometry and the material distribution. The F-Rep for geometric object *G* ⊆ *Ω*, where *Ω* is the modeling space, can be expressed as

$$ G=\left\{X|X=\left({x}_1,\cdots, {x}_n\right)\in \varOmega \subseteq {E}^n,F(X)\ge 0\right\} $$

Here, *F* : *X* → *R* is a continuous real-valued function of point sets that is at least *C*^0^ continuous, where the vertices satisfying *F*(*x*) > 0 belongs to the inside of the object, the boundary of the object corresponds to the vertices satisfying *F*(*x*) = 0, and the points with *F*(*x*) < 0 belong to the outside of the object. Hence, the entire point set can be represented by *F*. By a procedure traversing a tree structure with primitives in the leaves and operations in the nodes of the constructive tree, the function value of F-Rep at any given point can be evaluated and guarantee the closure property of the representation.

When considering heterogeneous materials, the F-Rep could be utilized to represent material distributions as well, where the generic model is a set of values embedded into a space of a proper dimension. Each point of the modeling space *Ω* ⊆ *E*^*n*^ is mapped to the attribute value set *N*_*j*_. Then, the F-Rep for the material attribute can be symbolically described as

$$ M=\left\{\Big({n}_j\in {N}_j|{n}_j={S}_j(X),X=\left({x}_1,\cdots, {x}_n\right)\in \varOmega \right\} $$

where *S*_*j*_ : *Ω* → *N*_*j*_ is an attribute real-valued defining function of point sets. Depending on a particular attribute, *S*_*j*_(*X*) can be either *C*^*k*^, *k* ≥ 0 or even not necessarily continuous at all. Note that different attributes can have both the same and different domains and value spaces, and there are no restrictions on operations over attributes. This indicates that it is possible to add or subtract values for attributes having “the same type”. Hence, one can easily deal with multi-component attributes, such as color composed of red, green, blue components. In ref. [71], the F-Rep provided a rigorous, concise and compact framework to specify, edit and analyze point sets and their material compositions. More importantly, F-Rep could also facilitates constructive modeling of complex objects from simple primitives in a similar fashion as the classical CSG representation.

Since implicit function based models allow to combine a geometric shape, material attribute, and other required attributes in one single description, and no conversion is needed. It is friendly to AM techniques. In addition, as it is typically specified procedurally, very little data could be used and the design procedure could be parallel computing.

### Composite model

When modeling generic objects with complex material distributions, two or more different material distribution types might occur in different portions of the same object simultaneously. Inspired by the idea of spatial partitions, composite models are suitable to represent such objects. In theory, a composite model is a collection of sub-objects, where each sub-object belongs to a specific material domain [72]. There are two different representations of the composite model, assembly model and cellular model.

**Fig. 8:**
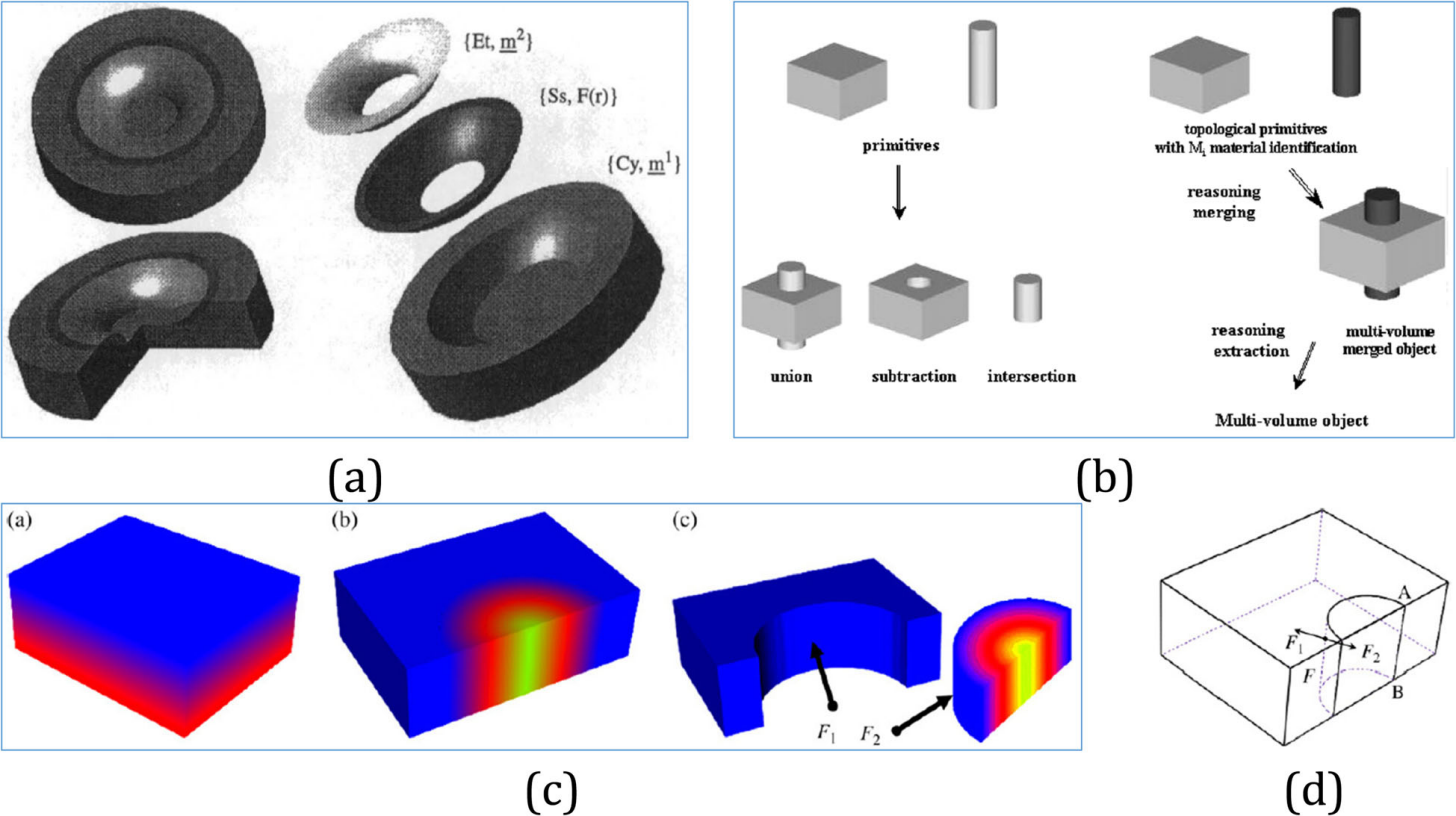
**a** Heterogeneous object made of simple material distribution sampled with the composition changing linearly from one material region to another [73]. **b** Comparison of conventional (left) and reasoning (right) boolean operations for heterogeneous objects modeling [74]. **c** When hybrid homogeneous, unidirectional or trivariate FGM distributions might occur in the same part, composite models are ideal to represent such objects. With the cellular model in (**d**), the delimiting face is no longer separately represented as shown in (**c**) [1]

Intuitively, the assembly model represents entire heterogeneous object as an assembly, and each sub-object corresponds to a unique material distribution. One constructive approach detects the existing primitives and generates spatial partitions from the boundaries of these primitives. As shown in Fig. 8a, b, some necessary operations, such as the regularized difference, intersection, and union were introduced in refs. [73, 74]. Based on a feature-based scheme, another possible approach could decompose the object directly into separate interconnected components [75]. Despite assembly model is easy to understand and use, the implementation of regularized boolean operations in spatial partitions could lead to some unnecessary operations or over-segmented 3D regions [76]; in addition, heavy redundancy and low consistency of the data may inherit some problems [48].

Compared with the assembly models, cellular models construct and maintain spatial partitions via non-manifold cellular representations that typically require more complicated data structures and algorithms. In such cellular representation, some topological elements should be distinguished from reference uses. As an example, any two boundaries share the same boundary element called co-boundaries, where each could obtain one of the two sides that oriented with respect to the geometrical structure. Through such type of co-boundaries, the cellular model with non-manifold B-reps can maintain the data redundancy to a minimum [77]. For heterogeneous objects, the very first cellular representation was proposed in ref. [78], which subdivided spatial partitions using a set of surface patches as delimitation boundaries. Recently, a similar approach was successfully applied in bio-modeling of heterogeneous bones [79]. By utilizing non-regularized boolean operators in ref. [48], the cellular geometries were constructed, and associated attribute was introduced based on the reasoning for modeling smooth inter-cell gradations.

By incorporating both assembly models and cellular models, hybrid composite models can represent objects with more complicated material distributions, and the merits of each representation can be fully exploited as shown in Fig. 8c, d. In a manner similar to the volume-mesh-based representation, a hybrid cellular functional model was proposed to represent complex heterogeneous objects [80]. Both the geometries and the material attributes were modeled with independent cellular-F-Reps. Therefore, the representational capacity could be fully leveraged to various heterogeneous objects. Whereas, hybrid composite models are mostly case-specific; the characteristics of compactness, efficiency and accuracy are all dependent on the involved component models.

### Complex topology structures

Most of complex topology structures are the nonzero genus geometries consist of numerous holes or voids, including lightweight structures, porous scaffolds, and lattice structures. The complicated topology is very common in the natural environment and reaps great benefits of the properties. For instance, the introduction of numerous holes could significantly reduce the weight of the model; in tissue engineering, the porous features of complex topology structures are suitable to human implants or scaffolds; some multi-functional targets can be achieved by adjusting the parameters of the structures [81]. Although complex topology structures have numerous advantages, they are difficult to fabricate via the traditional manufacturing processes. As the state-of-the-art technology, AM can fabricate these complex topology structures layer by layer with diverse materials.

**Fig. 9:**
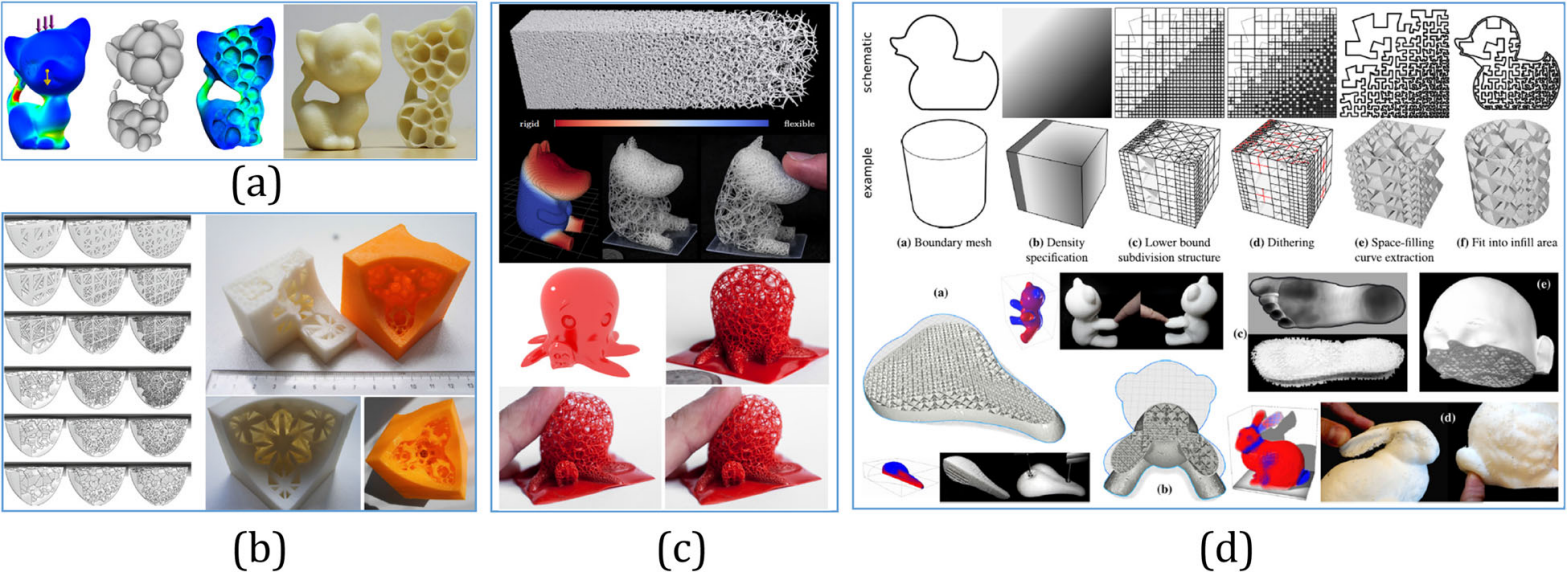
**a** Voronoi-diagram-based honeycomb-cell structure [82]. **b** Parameterized adaptive infill primal and/or dual cellular structure to hollow the solid models [83]. **c** Voronoi-based foam structure with controlled isotropic elastic behavior [84]. **d** Self-supporting foam structure based on a space-filling surface with spatially varying subdivision levels [85]

By considering the emergence of low-cost requirement in AM, Lu et al. [82] introduced a Voronoi diagram based honeycomb-cell structure as shown in Fig. 9a, which has minimal material cost while providing strength in tension. As shown in Fig. 9b, given a volume boundary, Sá et al. [83] presented an adaptive voids algorithm that can automatically generate a parameterized adaptive infill primal and/or dual cellular structure to hollow the solid models. In contrast to the hollowing-based methods, the foam geometry has microstructures at the scale of tens of microns that change the physical properties of objects and make them lighter or more flexible. As shown in Fig. 9c, Martínez et al. [84] used the Voronoi foams to design and generate microstructures with controlled isotropic elastic behavior. Then, for further improving the degree of design freedom, an orthotropic k-nearest foam design method was presented in ref. [86], where the elasticity of the constructed structures could be independently controlled along different directions. In ref. [87], the microstructures yielded a wide range of elastic behaviors from isotropic to orthotropic and the resulting foam geometry could be spatially graded. To design structures with spatially graded material properties, Kuipers et al. [88] proposed a self-supporting foam structure, called *CrossFill*, which is a space-filling surface that enables spatially varying subdivision levels in Fig. 9d.

**Fig. 10:**
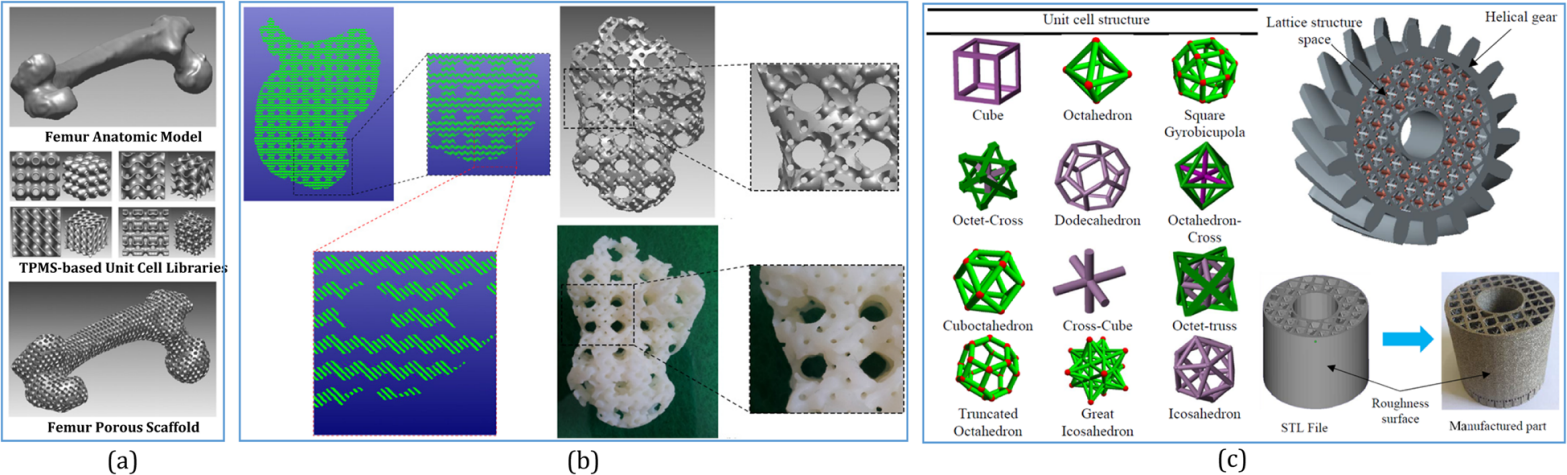
**a** Utilizing TPMS-based unit cell libraries to generate porous scaffolds from the subdivided hexahedral elements [89]. **b** The generated TPMS scaffolds could be intersected with TPMS libraries again by the distance field to generate hierarchical TPMS scaffolds [90]. **c** For any complex models, diverse unit cells can be selected or even combined to design the ideal lattice structures [91]

For cell multiplication in tissue engineering, porous scaffold has high porosity with interconnected and non-tortuous pores, which is an essential support to supply sufficient space for cell proliferation or nutrition transportation. As an implicit surface with intricate structures, the triply periodic minimal surface (TPMS) provides a concise description for various physical materials. In ref. [85], three type of TPMS were utilized to fabricate cellular materials (CM), which are Schwarz Primitive, Schoen IWP, and Neovius. It was observed that the TPMS-CMs show superior properties when compared with other cellular structures. Neovius-CM and IWP-CM have a similar mechanical response, showing higher stiffness and strength than the Primitive-CM. Thus, TPMS-CMs are promising candidates for various technological applications. As shown in Fig. 10a, for designing tissue-engineering scaffolds in ref. [89], after generating the hexahedral elements, the unit cell libraries composed of TPMS are mapped into the subdivided hexahedral elements, but parameter values related to the porosity and architecture type are simply allocated to the corner nodes. After applying the boolean operations of the anatomical model and TPMS-based unit cell libraries, defect-free porous scaffolds with complicated microstructures can be generated by combining the TPMS with the designed external shape efficiently [92]. Then, the generated TPMS scaffolds could be intersected with TPMS libraries again by the distance field to generate hierarchical TPMS scaffolds as shown in Fig. 10b [90]. To design both the external shape and internal architecture of scaffolds conveniently, the geometry structure in ref. [93] was defined by solid T-spline, and the internal complex porous structures were designed by the combination of internal control points and TPMS. By inheriting the local refinement algorithm from T-spline, the scaffold could be locally modified without changing the overall shape.

As another typical kind of complex topology structure, the lattice structure refers to a type of CM that can be considered a hierarchy of different cellular structure elements. By tailoring the material, heterogeneous lattice structures can be optimized to satisfy specific functional requirements, which indicate that the mechanical properties are more flexibly controlled. Based on their degree of order of the lattice frame, lattice structures are classified into three categories: randomized lattice structures, periodic lattice structure, and pseudo-periodic lattice structures [94]. As shown in Fig. 10c, the most common lattice units have been summarized [91]. For any complex models with various mechanical or other physical requirements, diverse unit cells can be selected or even combined to design the ideal lattice structures with the boolean operations.

## Process planning for heterogeneous objects in AM

AM is well known for printing 2D layers up to a 3D model regardless of its complicated geometrical structure and interior material distributions. Although there are various processes, the fundamental process planning steps are similar: part orientation, slicing the CAD models into 2D layers, and generating the scan path of each layer, which depends on the machine being used.

### Part orientation

Part orientation in AM hugely affects the fabricated product in many ways, such as mechanical strength, surface smoothness, printing time, and the amount of the support structure. When selecting a suitable method of orienting, a study of various types of methods indicates that part orientation can be categorized based on the types of features-both external and internal features. Furthermore, Mankame et al. [95] presented the issues related to the orientation and stacking of flat parts based on their internal features.

**Fig. 11:**
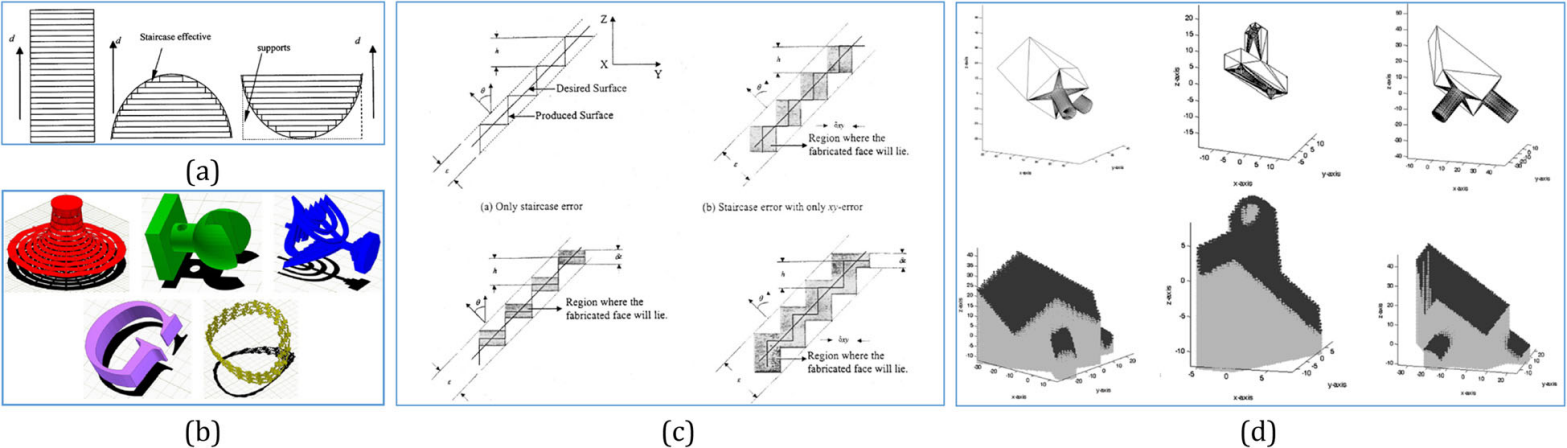
**a** Effect parameters of build orientation [98]. **b** The models in their computed optimal orientations with the cast shadow of very small areas [99]. **c** Effect of *xy*-error and *z*-error on the flatness error [100]. **d** Optimal orientation for minimum support structures [101]

Some related studies concentrate on computing the effect of the part orientation on a single property. For example, the work in ref. [96] selected a near-optimal orientation based only on minimal build time for a shape deposition manufacturing system. This method first represents all the candidate orientations among the unit sphere; then, partitions the unit sphere into smaller spherical polygons and assembles solutions within every spherical polygon to determine the final build orientation. Another common property examined is the volume of support materials. By considering convex models, Agarwal and Desikan [97] computed an orientation that minimizes the surface area of contact between 3D model and the support structure. As shown in Fig. 11 b, d, refs. [99, 101] were applied to calculate the volume of support materials directly for given orientations, and determined the best overall orientation that minimizes the volume of support materials.

When encountering the volumetric error in part orientation, a part-build orientation system was developed in ref. [102] to minimize the errors caused by the staircase effect in AM. This methodology involves a primitive volume approach, which assumes a complex geometry as a combination of basic primitive volumes. In ref. [103], the object was first sliced with horizontal planes; the volumetric error of each layer was then computed based on the resulting contours of each layer. After different orientations by rotation about user-specified axes, the best orientation with the least amount of volumetric error could be recommended. To investigate the relation between cylindricity tolerances, Paul and Anand [104] developed a graphical technique for calculating the optimal build orientation for a part with multiple cylindrical features.

By considering various orientation dependent properties together, the work in ref. [105] evaluated model height, support volume and surface accuracy and determined the best printing orientation with a cost comparison method. Another work similar to ref. [105] was conducted in ref. [106], which proposed an expert system tool that interacts with a user to select the best orientation. The build orientation in Fig. 11a was optimized based on the joint considerations of the staircase effect, support area, and production time together [98]. In ref. [107], computational quality metrics were proposed to quantify build time, material strength, and surface approximation error as functions of part orientation. For an AM process, these measures can be used to evaluate how significant orientation is, and to provide physically based models for the objective functions used to optimize orientation. Another two-step approach was produced with flatness requirements on the planar faces of a part to analyze the manufacturing feasibility of the parts [100]. As shown in Fig. 11c, each specified tolerance, such as staircase-errors, *xy*-errors, and *z*-errors, is analyzed and the set of feasible build directions that satisfies these tolerances is identified. Then, all sets of feasible build directions are intersected to identify build directions that can satisfy all the tolerance requirements. If such build directions exist, the object is considered manufacturable; otherwise, the part is considered non-manufacturable.

### Slicing methods

As an important procedure in AM, slicing converts the models into a collection of layers that can then be fabricated. In the literature, research on the slicing process can be divided into two main categories: slicing tessellated CAD models and the direct slicing method. Tessellated representation has several advantages such as easy implementation, flexible capability in orienting models and adding supporting structures. However, tessellated representation lacks high-order continuity and requires many elements to represent complex geometric details. The direct slicing method has been extensively studied to be directly applied to exact B-reps. It can reduce the pre-preparation time without involving the tessellation procedure and maintain the model integrity from the original representation to the sliced results and even the final product.

Adaptive slicing is an important computational task required in the AM process. The number of slices significantly affects the time required for fabricating, whereas their thicknesses affect the error. It is ideal to determine an optimal trade-off between the fabrication time and the surface quality. Assuming a discrete setting in ref. [108], the error was measured as the number of voxels incorrectly assigned due to slicing. Then, for any given set of available slice heights and a shape, optimal slicing can be achieved with minimal error. The study in ref. [109] generated slicing plans using a new metric profile that can characterize the global deviation errors along the building direction and determine the best slicing plan using dynamic programming. To overcome the staircase effect, Etienne et al. [110] curved the layers, making them either follow the natural slope of the input surface or otherwise; and then intersecting them with surfaces at a steeper angle, thereby improving the sampling quality.

Direct slicing of CAD models was firstly implemented with uniform slice thickness [111]. By tracing surface contours and cutting vectors, the model defined by multiple B-spline surfaces was directly sliced [112], and different cusp heights could be imposed on different surfaces [113]. Based on the concept of limited area deviation [114], a direct slicing algorithm was implemented using AutoCAD. For rapid prototyping, an adaptive approach was presented with non-uniform cusp heights independent of CAD systems [115]. Based on an NURBS model, a ray-tracing method was developed for direct slicing [116]. B-spline surfaces were constructed from a point cloud and were then sliced [117]. A global adaptive direct slicing technique [118] was proposed for NURBS-based sculptured surfaces. In ref. [119], the desired surface was defined parametrically via NURBS and then sliced with equally spaced height. With T-spline techniques, the direct slicing method becomes more suitable for manufacturing sophisticated freeform surfaces [120].

**Fig. 12:**
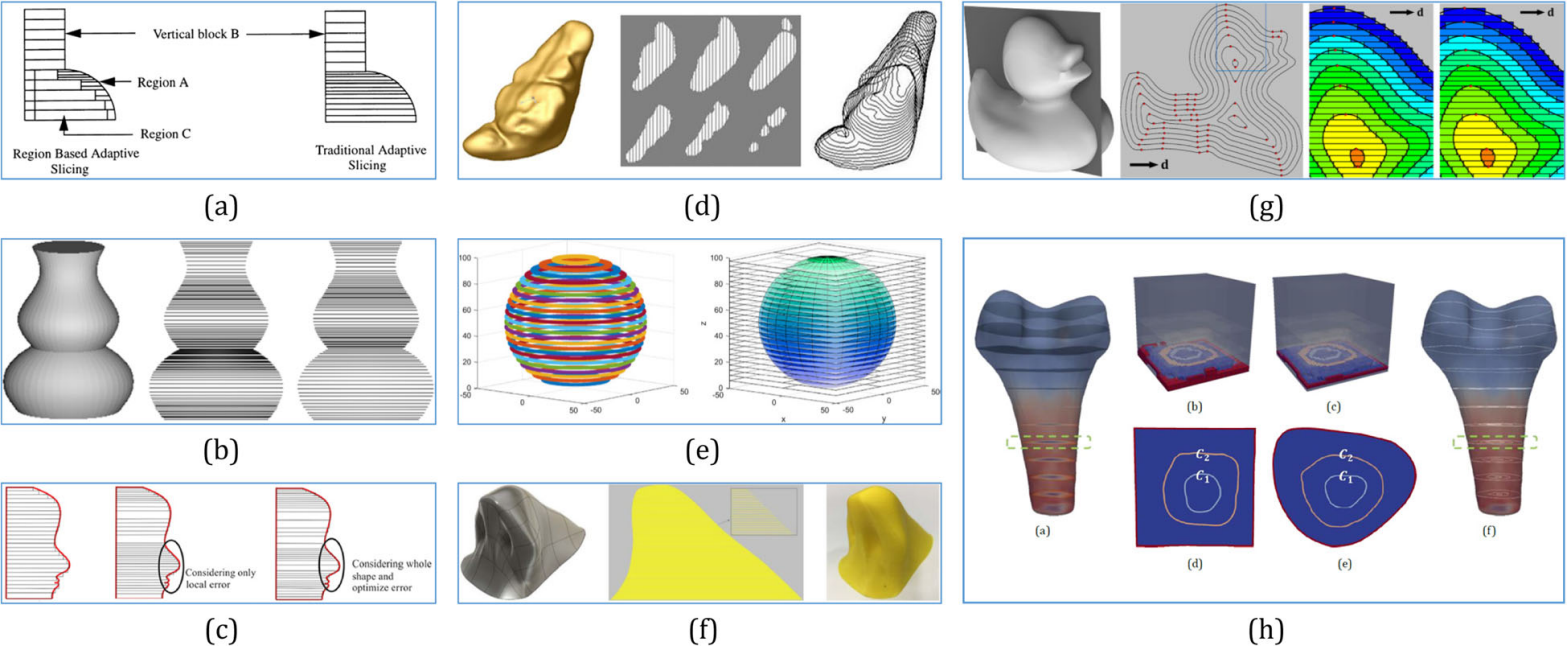
Some current slicing methods: Adaptive slicing results (**a**) [113], (**b**) [115] and (**c**) [118]; direct slicing results of NURBS models (**d**) [117], (**e**) [120], and T-spline models (**f**) [120]; direct slicing results of trivariate NURBS models (**g**) [64], and trivariate T-splines (**h**) [121]

As shown in Fig. 12a-f, although all these direct slicing methods have generated precise slicing contours from different surface representations, they only represent the boundary surface and do not carry any interior information such as material distribution and density variation. Heterogeneous solids are highly preferable for designing and manufacturing sophisticated models, such as biomedical objects with multiple biomaterials. A framework for the modeling of heterogeneous objects in terms of trivariate B-spline functions was proposed in ref. [64]. As shown in Fig. 12g, the B-spline volume and the associated attribute data were directly sliced without their conversion to the SLA format, resulting in a toolpath with fewer errors. To slice heterogeneous solids directly, a slicing algorithm using octree-based subdivision and trivariate T-splines was presented in ref. [121], which can be applied to the FDM technology.

### Path planning

**Fig. 13:**
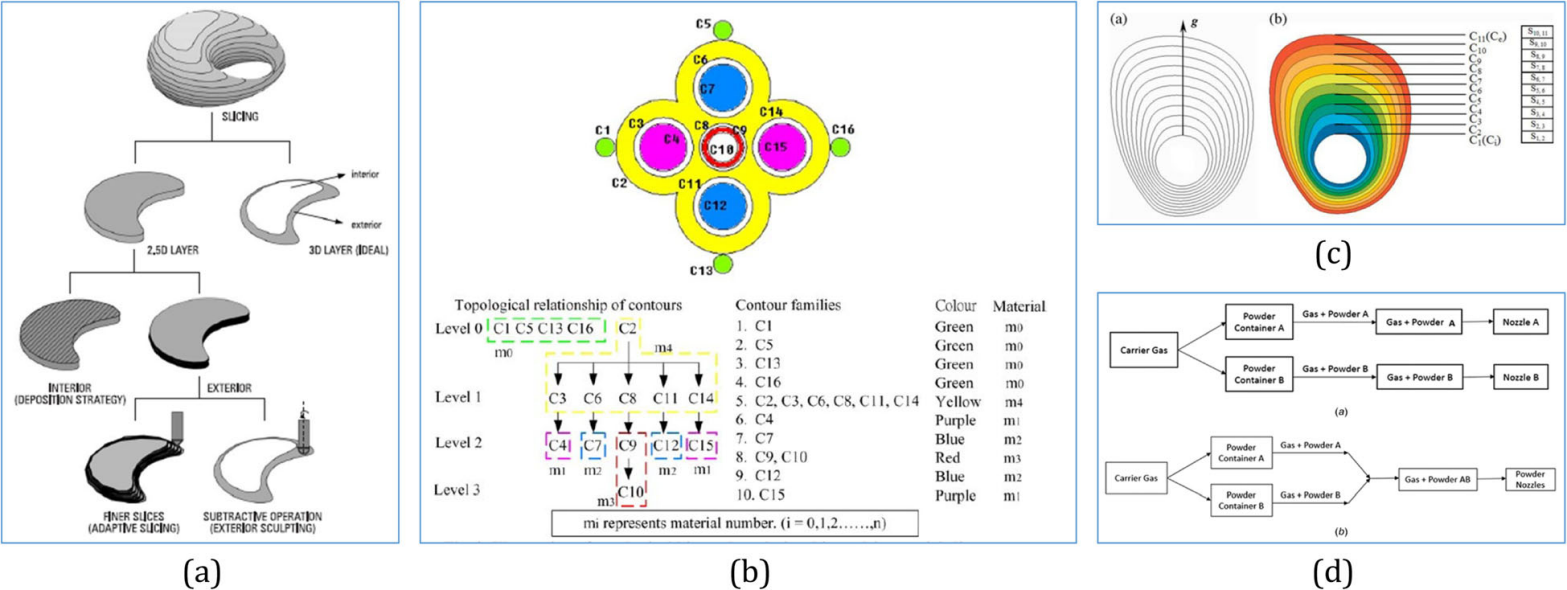
**a** General process planning in additive manufacturing [122]. **b** Illustration of topological hierarchy relationship of multi-material slice contours [123]. **c** EDO approach for the representation of sub-regional FGMs and then plan toolpath for fabricating [124]. **d** Two AM-based schemes for heterogeneous objects with FGMs [125]

As shown in Fig. 13a, toolpath planning for each single layer is mainly concerned with the strategy for filling the 2D surface. Typical region-filling toolpaths employ fixed strategies such as zigzag, linear, and contoured toolpaths. However, without controlling the composition of the deposited material, these toolpaths may not work for heterogeneous material objects [122].

For fabricating objects with discrete multiple materials, the way to build is to import several geometry parts into the system and partition them as separate material compositions. A simulation system for multi-material layered manufacturing was introduced in ref. [126], which planned a single toolpath per a single material and then integrated the toolpaths for each material into a single multi-material toolpath. However, this method could only process relatively simple objects, such as cylinder and cube, and only be used on extrusion-based machines. As shown in Fig. 13b, a multi-material virtual prototyping system used a topological hierarchy-sorting algorithm that processes the hierarchy relationship of complex multi-material slice contours [123]. Consequently, subsequently arranged contours could control the nozzles to move and deposit the selected materials at the appropriate contours by avoiding redundant back-and-forth movements. Another concurrent toolpath planning algorithm for multi-material layered manufacturing was presented in ref. [127]. To improve the fabrication efficiency of relatively large and complex prototypes further, this approach incorporates a decoupled motion-planning technique for multiple moving objects with a collision detection algorithm and a dynamic priority assignment scheme. However, it only adopts up-and-down zigzags for internal contour filling, and it would be useful to include the spiral contour-filling mode.

By using direct energy deposition processes, the feed rate from different nozzles can be controlled to mix the material composition during the printing process, which is capable to switch material category and print objects with gradually varied materials. Furthermore, it is necessary to plan a global automatized toolpath with the right material deposition strategy on each single spot. Such a toolpath planning strategy was proposed in ref. [128]. This algorithm discretizes the gradual material distribution in each layer into sub-regions with homogeneous material. Then, a sequential toolpath for each sub-region is generated separately for the fabrication of functionally graded multi-material objects. Equal distance offset (EDO) [124] is another novel approach for the representation and process planning for FGM objects. As shown in Fig. 13c, in EDO, a neutral arbitrary 3D CAD model is adaptively sliced into a series of 2D layers. Within each layer, 2D material gradients are designed by subdividing the 2D shape into several sub-regions enclosed by iso-composition contours. Using this approach, an arbitrary-shaped 3D FGM object with linear or non-linear composition gradients can be represented and fabricated via suitable machines. Without discretization of the material distribution, two path strategies, raster and spiral paths, were compared with performance indexes, and a good correlation was demonstrated between the simulated and deposited material distributions [129]. As a general approach to handle the range of heterogeneous parts from multi-materials to FGM distributions, an automate toolpath planning approach was presented by jointly considering the process parameters together, such as traveling time between two connecting positions, material switching time for discrete materials, and the stabilizing time [125].

## Conclusion and open topics

In this study, we reviewed AM-based processes for the fabrication of heterogeneous material objects, which is a promising area of research and development. We classify the representation techniques depending on the different expression or data structure employed. The state-of-the-art process planning pipeline for AM was also reviewed via different existing solutions for part orientation, slicing methods, and path planning processes. The possible future directions and challenges are discussed in this section.

- In current state, suitable representations for objects with complex heterogeneities is non-trivial to approach. Most existing approaches claim the representation of heterogeneous material objects via traditional geometry-based schemes. However, the resulting material models are usually more complicated than the geometry, even for simple distribution. The representation for irregular material distributions should be given special attention and be independent on geometry.
- Unlike current heterogeneous object modeling techniques that focus on space-dependent material heterogeneities, dynamic heterogeneous objects incorporate time phase into material heterogeneities. For example, based on the B-spline-based modeling methods, Qian and Dutta [57] extended the diffusion equation to manipulate time-dependent heterogeneous materials; the framework in ref. [80] exhibits inherent multidimensionality from a hybrid cellular functional model and deals naturally with time-dependent properties to model complex dynamic heterogeneous objects. By considering time, dynamic heterogeneity modeling could have more realistic applications in many interesting and important areas. Therefore, the attribute properties of arbitrary nature (material, photometric, physical, statistical, etc.) need new representation schemes, supporting data structures, algorithms, and methods for such innovations to be maintained.
- The study in in CAD, CAE, and CAM fields somehow has generally distinct gap. With the development of IGA, this gap may be bridged with some new functional expressions. In addition, the design and representation of heterogeneous material objects inherits inter-disciplinary connection in each of these fields. Moreover, compared with traditional manufacturing processes, AM-based systems, especially for their process-planning pipeline that require better control on process parameters, show the potential to connect studies in each area intrinsically. Therefore, an extensive AM-based study is required to build a system with highly controllable process parameters to obtain the desired performance and accuracy. This typical system should be robust to incorporate and account for special uncertainties to bridge the gap between the CAD, CAE, and CAM fields.

## Data Availability

Not applicable.

## Abbreviations

2D: 2 Dimensional
3D: 3 Dimensional
4D: 4 Dimensional
AM: Additive manufacturing
B-rep: Boundary representation
CAD: Computer aided design
CAE: Computer aided engineering
CAM: Computer aided manufacturing
CM: Cellular materials
CSG: Constructive solid geometry
CT: Computerized tomography
EDO: Equal distance offset
FDM: Fused deposition modeling
FGM: Functionally graded material
F-Rep: Functional representation
IGA: Isogeometric analysis
SLA: Stereo-lithography
TPMS: Triply periodic minimal surface
